# Semantic Description of Quality of Data in Sensor Networks

**DOI:** 10.3390/s21196462

**Published:** 2021-09-28

**Authors:** Anupam Prasad Vedurmudi, Julia Neumann, Maximilian Gruber, Sascha Eichstädt

**Affiliations:** Physikalisch-Technische Bundesanstalt, 10587 Berlin, Germany; julia.neumann@ptb.de (J.N.); maximilian.gruber@ptb.de (M.G.); sascha.eichstaedt@ptb.de (S.E.)

**Keywords:** Internet of Things, semantics, ontology, measurement uncertainty, calibration

## Abstract

The annotation of sensor data with semantic metadata is essential to the goals of automation and interoperability in the context of Industry 4.0. In this contribution, we outline a semantic description of quality of data in sensor networks in terms of indicators, metrics and interpretations. The concepts thus defined are consolidated into an ontology that describes quality of data metainformation in heterogeneous sensor networks and methods for the determination of corresponding quality of data dimensions are outlined. By incorporating support for sensor calibration models and measurement uncertainty via a previously derived ontology, a conformity with metrological requirements for sensor data is ensured. A quality description for a calibrated sensor generated using the resulting ontology is presented in the JSON-LD format using the battery level and calibration data as quality indicators. Finally, the general applicability of the model is demonstrated using a series of competency questions.

## 1. Introduction

The increasing automation of manufacturing and the concurrent use of interconnected cyber-physical systems, large-scale heterogeneous sensor networks as well as machine learning methods are key aspects of the Industrial Internet of Things, or IIoT, paradigm [1]. They pose a unique set of challenges which are exemplified by the Industry 4.0 goals of interoperability and decentralization [2]. As one of the primary interfaces between the physical and digital worlds, sensors play a central role in IoT by providing data typically in the form of numerical values corresponding to a given physical quantity; in other words, a measurement. A key concept in IoT is the combination of multiple interconnected measuring instruments to create a sensor network that functions as a distributed measuring system. Sensor networks are of particular importance in industrial environments and, in general, can be either homogeneous or heterogeneous in nature, i.e., with constituent sensors that measure, respectively, the same or different physical quantity/-ies. In the context of these endeavors, the automated transmission, analysis and processing of sensor data are key components at every level in IIoT-systems. Consequently, a deeper understanding of the quality of data (QoD) in conjunction with the means to make such information available to all data users involved—be they applications, human actors or even other sensors—is of utmost importance.

One example of a fundamental quantity that can be considered as a quality metric ascribed to a sensor is the measurement uncertainty—“a parameter associated with the result of a measurement that characterizes the dispersion of the values that could reasonably be attributed to the measurand” [3] (p. 14), i.e. the physical quantity being measured. The measurement uncertainty is determined by means of a calibration such that each measurement result is related to the SI-unit reference through an unbroken chain of calibrations, resulting in a form of quality assurance referred to as metrological traceability. The incorporation of metrological information such as the measurement uncertainty in IoT, although in its relative infancy, is an active area of research [4]. The term calibration in a metrological sense is defined according to the International Vocabulary of Metrology (VIM, fr: Vocabulaire International de Métrologie) [5] as “an operation that, under specified conditions, in a first step, establishes a relation between the quantity values with measurement uncertainties provided by measurement standards and corresponding indications with associated measurement uncertainties and, in a second step, uses this information to establish a relation for obtaining a measurement result from an indication”. In addition to the uncertainty, other data relating to the calibration (such as an identifier for the sensor, the units and the name of the laboratory) can be stored in a digital calibration certificate (DCC, [6]) for use in digital infrastructures.

Calibrations are typically carried out in strictly controlled laboratory environments. Sensors, on the other hand, are deployed in diverse environments and operating conditions that may influence the QoD in various ways. For instance, in the case of wireless sensor networks, unstable connections, congestion, environmental interference, and malicious attacks can result in missing data [7]. In battery powered sensors, there is necessarily a trade-off between power consumption and performance [8]. As a result, an exhausted or damaged battery can also be detrimental to the quality of measurements produced by a sensor. Another common issue is drift [9], where sensor readings gradually stray from the true value with time due to the degradation of the electronics. Other anomalies common to sensor readings are spikes—sharp changes between successive measurements deviating from normal sensor behavior –, freezing or constant values [10] and stuck-at-zero errors resulting from a dead sensor. These problems are exacerbated in typical IoT settings, since deploying a large number of high-cost sensors that individually conform to desired accuracy criteria is not feasible in many applications, particularly in those that require large and dense sensor networks [11]. As sensors in an IoT network typically have small batteries and limited working memory, correcting faults by re-transmitting missing data over the network would consume excessive amounts of power and computational resources while increasing inaccuracies due to the delays introduced [7].

A more general description of the quality of data in sensor networks thus requires the consideration of the aforementioned issues in addition to the measurement uncertainty. In order to generate such QoD knowledge, sensors should ideally be able to communicate information about themselves and their environment. The deployment of sensors endowed with a level of “smartness” [12,13] is a key to enabling the communication of useful metainformation in IoT environments. Such smart sensors are capable of a certain amount of pre-processing and possess a degree of awareness regarding their surroundings and hence the ability to communicate metainformation about themselves and their measurement tasks in addition to the actual measured values. Effectively communicating such self-information in an independent manner enables reliable data analysis and error-handling [14] and thus helps to improve interoperability in IoT systems. The notion of metrological traceability was extended to networks of smart sensors in IoT in [8].

The goal of the present work is to formulate and justify a consistent, reusable, and modular description of quality of data in sensor networks, which we define as the “fitness for use” [15] of given data with respect to a specified purpose. We achieve this goal by first outlining a semantic description of QoD in sensor networks with an emphasis placed on generality and flexibility. We do so by first presenting a general data model for data quality in sensor networks. We then use this knowledge to construct an ontology by extending the semantic description and by merging concepts from relevant external ontologies to include the necessary relationships between classes and thus simultaneously fulfill the IIoT requirements of machine readability and interpretability. In Section 2 we discuss the details of QoD in sensor networks and construct a basic scheme for its semantic representation. In particular, the QoD description is separated hierarchically into indicators, metrics, and interpretations, with illustrative examples provided for each category. The semantic representation thus developed is extended in Section 3 in order to construct a formal, machine-readable, and machine-interpretable description of QoD in the form of an ontology which takes into account the relationships between the concepts previously defined and integrates entities from established ontologies. Finally, in Section 4, the model is evaluated by formulating a QoD description for a real-world use case. In particular, a potential representation of QoD in sensor networks using the JSON-LD data format is presented along with a series of competency questions that demonstrate the applicability of the ontology.

## 2. Semantic Description of QoD in Sensor Networks

The automatic processing and interpretation of raw data corresponding to sensor measurements is generally not practicable. This is due to the fact that for a naive receiver of sensor data (for example, an edge device like a router) with no knowledge of the attributes of the sensor, raw sensor data by itself is merely a stream of numbers. However, by augmenting the measurement values with meaningful metadata, devices in a sensor network that process data from a given sensor would be able to automatically interpret its raw data. Such information ascribes “meaning” to raw data and is referred to as semantic metadata. The rules and models necessary to enable a formal representation and interpretation of such metadata, in conjunction with the means to exchange and process them, are provided by semantic technologies. The semantic web community [16,17,18] has been active in the development and support of technology standards, methods and tools to enable sensors to automatically provide information about themselves and their environment. Quality of data (QoD), or data quality, is an example of such metainformation and, in the present context, is defined as the “fitness for use” [15] of sensor data for a given purpose. For sensors to be able to automatically communicate and interpret metainformation relating to data quality, a systematic description of the involved concepts is necessary.

By requiring the intended use of data to be central to the definition of QoD, an emphasis is placed on its inherent subjectivity. The assessment criteria of QoD are dependent both on the history or provenance of the data and on the context in which it is being used [19]. Typically, the quality of a single sensor’s measurements can be assessed on the basis of a set of indicators that includes, but is not limited to, accuracy, completeness and timeliness. The aforementioned indicators can be directly quantified by means of an appropriate metric. For instance, the completeness of a series of sensor readings within a particular time window can be computed as the ratio of the number of non-missing readings to the total number of readings [20]. Other indicators are similarly provided with a means to quantify the QoD. The resulting “score” allows the user of the data to make judgments regarding its fitness. The proposed data model for QoD consists of four main components:

- an abstract measuring “System”;
- a QoD indicator corresponding to a particular sensor or data property;
- a metric describing an assessment procedure for the indicator;
- an interpretation of the metric.

**Figure 1:**
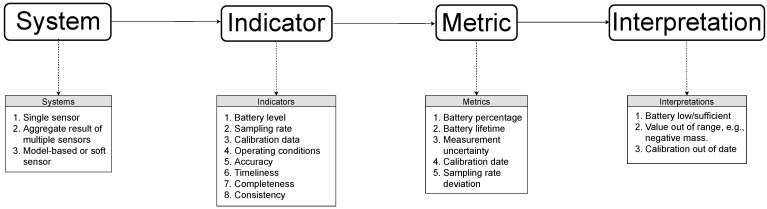
Overview of the basic scheme for representing QoD in sensor networks. Each abstract “system” has at least one QoD indicator which is in turn assessed using a particular metric. The metric is then associated with an interpretation that allows automatic processing of the QoD information. Illustrative examples have been provided in the boxes below each component of the scheme.

By generalizing the objects under consideration to abstract measuring systems, an assessment of QoD is made possible both for physical sensors as well as for aggregates of multiple sensors and soft or model-based sensors [21]. Soft sensors are inferential estimators that provide “sensor-like” data based on a mathematical model applied to observations from single or multiple hardware sensors. The models may be derived using knowledge of the physical principles involved or through the use of machine learning methods [22]. The quality of data of such a virtual object in a sensor network is dependent on the QoD of the constituent sensors. Furthermore, each abstract “system” is associated with at least one QoD indicator which is in turn assessed using an appropriate metric. The metric is then provided with an interpretation allowing an automatic processing of the QoD information. The resulting scheme for representing QoD in sensor networks is shown along with illustrative examples for its different components in Figure 1.

### 2.1. QoD Indicators

A description of QoD for a given system would require us to assign one or more indicators (also referred to dimensions [23]) to it. In addition to data completeness, indicators like accuracy, timeliness, or consistency, as computed from its measurements in a given time window, can be used to describe the QoD of a sensor. Furthermore, numerically quantifiable properties such as sensor battery level and energy consumption, sampling rate and network bandwidth have non-trivial effects on the QoD that are impractical to compute in IoT setups given the limited processing power of the involved components. The communication of the data transfer rate is important, particularly in the case of sensors that are able to vary their data volume on demand. Including such quantities as QoD indicators would therefore be advantageous at the point of data acquisition. In order to ensure the traceability of the QoD metrics and indicators, the sensor ID should also be included as part of the QoD assessment. Although indicators such as calibration data and environmental conditions can be defined for physical sensors, this cannot be done meaningfully in the case of soft sensors. It is, however, possible to add the mathematical details of the used model or the train/test accuracy (in the case of ML-based sensors) to the QoD description. Some attributes that can serve as QoD indicators for sensors are [24]:

- Accuracy: The degree of “closeness” of the data with respect to the correct measurement of the physical phenomenon being observed. The accuracy of a new sensor in a network can be assessed by comparing it, for example, with a reference sensor or with an aggregate value from multiple sensors observing the same physical quantity.
- Completeness: A measure of the number of missing or null values. A high percentage of missing values from a sensor could stem from hardware or network issues and is bound to influence the usability of the data.
- Timeliness: A quality dimension expressing the currency or recentness of the data. Certain users or time-critical applications can use this indicator to assess the time delay between the measurement and the acquisition of data.
- Consistency: The degree to which data adheres to pre-defined criteria. For sensor data, the operating range is a common criterion. Another example would be to check for the consistency of the data according to its measurement principle, e.g., negative masses not allowed; cf. Figure 1.
- Battery level: The sensor battery level can be represented either as a percentage or as the remaining lifetime given the current rate of power consumption. As sensors tend to provide unstable readings towards the end of their battery lifetime [25], a low battery level also serves as a predictor for other QoD issues.
- Calibration data: Information such as the results of a calibration in the form of a measurement uncertainty as well as administrative metadata such as the place and date of calibration and the qualification of the person carrying out the calibration are included in a digital calibration certificate (DCC) [6].
- Operating conditions: Any description of a sensor’s behavior must normally include the specification of its operating conditions. Sensor calibrations, for instance, are performed under the specific temperature and humidity conditions experienced during sensor operation.
- Sampling rate: A sensor’s actual sampling rate can differ greatly from the value specified on its data sheet. For instance, a smart sensor may lower its own sampling rate in order to reduce power consumption.

### 2.2. QoD Metrics

In order to be able to make decisions based on QoD, an indicator first needs to be associated with a corresponding metric, which serves as a means to assess the quality “level” of that indicator [26]. In the present work, we define a metric as a mathematical object that assigns a score to a sensor with respect to a particular QoD indicator. In general, a given indicator can have more than one metric. For instance, the accuracy indicator can be associated with a metric defined in terms of a general distance function D(.,.), which takes the sensor reading $v_{n}$ received at time $t_{n}$ and a reference value $v_{n}^{\prime }$ of the measured quantity as arguments. The distance function $D(v_{n},v_{n}^{\prime })$ is zero when $v_{n}=v_{n}^{\prime }$ and positive otherwise. The resulting score $S_{A}$, calculated using the metric 1/(1+D(.,.)), is given by

(1) $$S_{A}=\frac{1}{1+D(v_{n},v_{n}^{\prime })}\;.$$

As a result, the computed score $S_{A}=1$ when the sensor measurement is exactly equal to the reference value of the quantity being measured and <1 otherwise. The choice of both the distance function and the reference sensor depends on the user requirements. Using the above metric, an application would be able to compare the accuracy of two sensors.

Similarly, a completeness metric can also be defined for a sensor in terms of the number of missing values in a given time window. For *N* data points received from a sensor in a time window $\left[t_{n},t_{n+N}\right]$ of which $n_{\mathrm{miss}}$ values are missing, the completeness metric results in a score given by the fraction [26]

(2) $$S_{\mathrm{com}}=1-\frac{n_{\mathrm{miss}}}{N}\;.$$

The above completeness metric thus has a maximum score of 1 when there are no missing values. The metric is thus defined as the fraction of missing values and results in a score $S_{\mathrm{com}}$ when $n_{\mathrm{miss}}$ out of *N* values are missing. In contrast to accuracy, a reference sensor is not needed to compute Equation (2) as each sensor reading is paired with a timestamp.

A possible metric for consistency is to check if the measured value lies within a particular interval specified in terms of a minimum and maximum value, say $x_{\min}$ and $x_{\max}$. An interval can either be closed, open, closed-open or open-closed according to whether it includes $x_{\min/\max}$. An example of the use of numerical intervals is a consistency indicator with the operating range as a criterion. Suppose that the measurement range of a pressure sensor is 0 MPa to 5 MPa, a sensor repeatedly returning values out of this range is not behaving consistently. A simple metric would be to assign a score of 1 to measured values between 0 MPa to 5 MPa and 0 otherwise. An example of a more involved metric that heavily penalizes negative pressures but allows high pressures to a certain extent can be defined as

(3) $$S_{\mathrm{con}}=M_{\mathrm{con}}\left(p\right)=\left\{\begin{array}{cc} 0 & \mathrm{if}\;p<0\\ 1 & \mathrm{if}\;0\leq p\leq 5\;\mathrm{MPa}\\ e^{1-.2p} & \mathrm{if}\;p>5\;\mathrm{MPa} \end{array}\right.\;.$$

The above measure returns a value of 0 for negative pressures, 1 for pressures within the allowed range and exponentially decreasing values above 5 MPa. This ensures that values are penalized more the further they exceed the upper limit.

A simple metric for timeliness is to compute the difference between the time $t_{A}$ at which an application receives a data point from a sensor and the timestamp $t_{D}$ of the data point itself $\left(t_{A}-t_{D}\right)$ and to assign a value 1 if it is below a particular threshold $T_{\max}$ and zero otherwise. A more involved metric [20] takes into account the response time $t_{R}$ of the sensor, i.e., the time delay between an observation being made and the sensor reading being sent out such that the timeliness score is given by

(4) $$S_{T}=M_{T}\left(t_{A}-t_{D}\right)=\left\{\begin{array}{cc} 1-\frac{t_{A}-t_{D}-t_{R}}{T_{\max}} & \mathrm{if}\;t_{A}-t_{D}\leq T_{\max}+t_{R}\\ 0 & \mathrm{otherwise} \end{array}\right.\;.$$

The above metric assigns a linearly decreasing timeliness score to values “newer” than $T_{\max}+t_{R}$ and 0 to values “older” than $T_{\max}+t_{R}$.

In contrast to the above examples, sensor attributes such as battery level, calibration data and operating conditions are indirect indicators of data quality, i.e., their metrics cannot be directly computed from the data. Moreover, administrative metadata such as the date of calibration, the accreditation status of the calibrating laboratory and the manufacturer ID can play a role in the QoD requirements for the end user even though their effect cannot be quantified directly. Administrative metadata in the present context refers to data that is ancillary to the main informational content of a given resource, but is nonetheless necessary to manage and use it. Providing end users with these values would enable them to make a suitable, requirements-based assessment of QoD. Quality metrics that can be defined in terms of calibration data are:

- Measurement uncertainty: The uncertainty on a calibration certificate can be directly incorporated into a quality description for a sensor. The “score” attributed would be the numerical value of the uncertainty along with the physical units.
- Recency: The “age” of the calibration or when the sensor was last calibrated. Given the natural wear of components, sensors which have been recently calibrated are by and large more trustworthy. The metric in this case would be the difference between the current timestamp and the timestamp corresponding to the calibration date.

The sensor battery level can be represented either as a percentage or as the remaining lifetime based on the current rate of power consumption. Specifying the remaining lifetime as a quality score also necessitates the specification of the time unit. A sensor’s actual sampling rate can differ greatly from the value specified on its data sheet. For instance, a smart sensor may lower its own sampling rate in order to reduce power consumption. A potential metric in this case is the deviation from the nominal sampling rate of the sensor. Because sensor calibrations are performed under strictly controlled environments, a description of a sensor’s behavior must usually contain the specification of that sensor’s operating conditions. A potential metric for a sensor specified to operate between temperatures $T_{\min}$ and $T_{\max}$ is 1 when the operating temperature lies between the aforementioned values and 0 when it lies outside. A more involved metric similar to the one defined in Equation (3) can also be formulated such that large deviations from operating conditions are heavily penalized. The necessary temperature measurements in this case are ideally available from a proximate sensor or one on the same platform. Similar metrics can be defined for ambient pressure and humidity.

### 2.3. QoD Interpretation

The concepts defined thus far encompass the description of various sensor and network properties that could serve as quality indicators, and metrics that describe mathematical objects and operations needed to assign a score to a given indicator. However, the results of such operations are by themselves not amenable to automatic processing. In order for the results of applying QoD metrics to sensor data to be machine interpretable, the notion of interpretation itself needs to be defined as a separate concept. In other words, the result of using a metric to compute a numerical “score” for a particular indicator needs to be accompanied by a semantic description of the score that appropriately categorizes the result and allows a receiver to ascertain whether the given data is “good”. Some potential interpretations corresponding to the results of a QoD assessment are:

- the sufficiency of the battery level with respect to certain requirements;
- the presence of drift beyond a certain threshold in sensor measurements;
- the calibration not being sufficiently up-to-date;
- the sensor operating in unsuitable environmental conditions.

In each of the above examples, at least one reference value representing the data receiver’s quality requirements is necessary. In the first two cases, this corresponds to a threshold value below which a battery level is deemed insufficient or critical, or a value above which a sensor is considered to have an unacceptable level of accuracy. Similarly, the calibration data of a sensor can be interpreted to be out-of-date based on a given reference time period. In certain cases, two or more reference values can be defined for a more detailed quality interpretation. For instance, the battery level can be further classified as “good”, “sufficient”, or “critical” given two reference values. A QoD interpretation thus augments the potentially complex result of an assessment using the metrics defined in Section 2.2 with a simpler characterization.

## 3. Ontologies in Sensor Networks

The semantic description of QoD established in the preceding section complements the ongoing work to formally describe semantic connections of concepts used to describe sensor networks. A common way to model such information in a flexible and machine-interpretable manner is by means of an ontology [27], i.e., a formal representation of a domain of knowledge. The main components of an ontological model are the individual classes belonging to a domain, the attributes or properties of these classes, and the relationships among class members. Ontologies further consist of axioms and class restrictions [28], which serve as a way to incorporate *a priori* domain knowledge into the ontology in the form of statements that are asserted to be true. A key focus of the semantic web community is the development of ontologies which formalize the annotation of sensor data with spatial, temporal, and thematic metadata [29]. Spatial metadata corresponds to the location information of a sensor and can be indicated either according to an absolute/geographical frame or a local/relative reference frame. Sensors mounted on a moving object like an automobile or a wearable device are typical examples of an application of the latter representation. Temporal metadata contains information regarding the time instant or interval when the sensor data was recorded, such as the timestamp indicating when the sensor measurement was taken. Thematic metadata refers to the description of the real world that is derived from sensor observations and cannot be covered by the first two metadata types. This often corresponds to domain-specific concepts such as the description of a machine component close to failure. Quality of data belongs to this category and can be considered a form of thematic metadata that is derived from sensor data analysis. By enriching the concepts developed in Section 2 with semantically expressive descriptions of attributes and inter-class relationships, a formal ontology for QoD can be developed.

Given their inherent flexibility, ontologies are particularly suited to the description of sensor networks. They can be merged with each other and constructively extended to include new or missing knowledge. A merged ontology, scal, which describes thematic metadata corresponding to sensor calibration information in conjunction with temporal and spatial metadata, was proposed in [30]. The ontology was later extended [31] to include the dynamic transfer behavior of sensors in a new ontology referred to as trans. In particular, tools such as the Semantic Sensor Network (SSN, [32,33]) and Sensor, Observation, Sampling and Actuation (SOSA, [34]) ontologies were used to model sensors and actuators along with their observations, the procedures involved, the studied features of interest and the samples used. The scal ontology established a method for storing sensor metadata in a machine-readable and -interpretable format by combining SSN and SOSA along with other ontologies and data models. By including calibration information in the ontology, essential metrological requirements were fulfilled. In addition to the SSN and SOSA ontologies, the sensor’s self-description was achieved by combining

1. the Digital SI (D-SI, [35]) data model to represent the observation values, units and uncertainties,
2. the *Ontology of Units of Measure and Related Concepts* (OM, [36]) along with ideas from the *Engineering Mathematics* (EngMath, [37]) ontology to represent physical quantities, their units and kinds and,
3. the *Geographic Query Language* (GeoSPARQL, [38]) for the geometric and topological location information.

**Figure 2:**
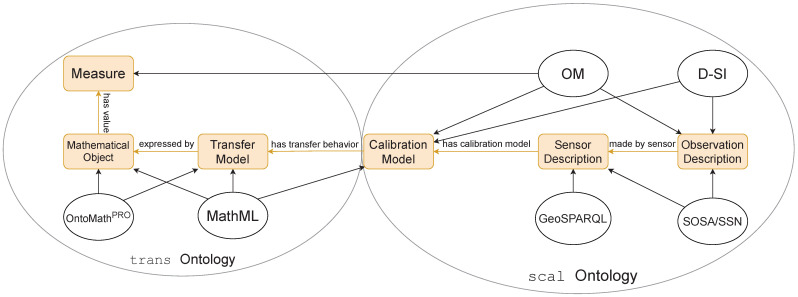
The merging scheme used to generate the trans [31] and scal [30] ontologies from external ontologies and data models. The “Transfer Model” class is an abstraction corresponding to the dynamic transfer behavior of sensors, which is expressed by objects belonging to the Mathematical Object class. The Measure class assigns numerical values to the model parameters.

Unlike SOSA, SSN, OM and GeoSPARQL, the D-SI model is not an ontology and cannot express the interconnections between concepts such as quantity, unit, or calibration model. It is, however, an indispensable part of the scal ontology as it covers aspects essential to metrology and traceability to SI units. Additionally, the semantic structure of the mathematical calibration model was described using MathML [39], while the temporal data was represented using the XML “dateTime” datatype in the format “YYYY-MM-DDThh:mm:ss[Z|(+|-)hh:mm]”, where Z refers to the time zone. The trans ontology extended the aforementioned model with concepts from the OntoMath${}^{\mathrm{PRO}}$ ontology [40] in order to model the mathematical details of the dynamic transfer behavior of sensors. The transfer behavior corresponding to the sensor calibration model is represented by the abstract TransferModel class and its constituent mathematical elements were modeled using the MathematicalObject class. The numerical values of the corresponding parameters (e.g., coefficients of a polynomial) were represented using the OM ontology and MathML as elements of type “measure” from the OM ontology which was extended using the D-SI model to include measurement uncertainties. The basic structures of the scal and trans ontologies are illustrated in Figure 2. “Properties” are entities that formalize relationships in ontologies. The two main kinds of properties are “object” properties that relate classes to one another, and “datatype” or “data” properties that relate classes to a “literal” datatype like an integer or a string. An example of an object property in the trans ontology is isExpressedBy which relates a calibration model to an individual of the MathematicalObject class. As a result, the parameters of models can be expressed as literals in the form of MathML, XML or T_E_X expressions. Typically, object and data properties are accompanied by restrictions [28]. The main data property in the trans ontology derives from the hasNumericalValue property of the OM ontology and places few restrictions on the target datatype. An element of an ontology is expressed via a prefix or namespace. For instance, trans:MathematicalObject refers to the MathematicalObject class in the trans ontology namespace. In the following, we construct an ontology that ascribes a semantically expressive structure to the QoD concepts discussed in Section 2.

### 3.1. The *qod* Ontology

As in the semantic description of the measurement and transfer behavior of sensors [30,31], the procedures and concepts necessary to describe data quality in sensor networks cannot be covered by a single ontological model. On the other hand, the inherent flexibility of ontologies allows us to combine different ontologies, data schemes and vocabularies to generate an appropriate description of QoD. Previous research [20] focused on extending the SSN ontology to model quality-of-sensing (QoS) attributes such as accuracy, timeliness, and completeness in individual sensors as well as on methods to compute these attributes. The QUAL-O ontology [41] describes quality assessment in sensor networks and was used in conjunction with the PROV-O [42] and SSN ontologies to perform a quality assessment on the observation of a temperature based on a given consistency metric by examining its provenance. The score corresponding to the consistency metric was calculated by comparing the measured temperature with average values for the given location and time of year. An additional namespace int covered the intent, i.e., the reason behind performing a given quality assessment. In contrast, the focus of the present work is to systematically describe the QoD itself such that both the QoD assessment and interpretation can be processed automatically.

**Figure 3:**
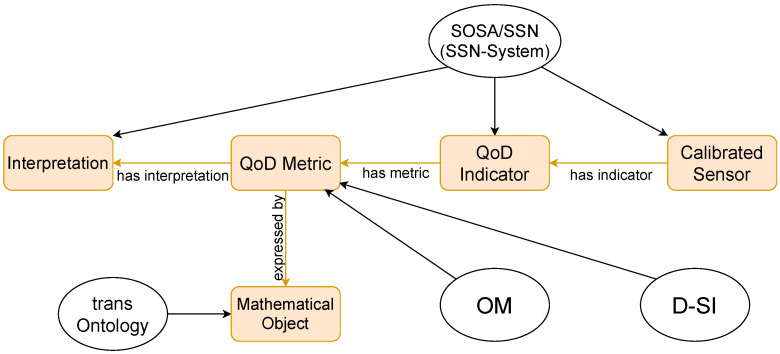
The qod ontology is constructed from a merge of the trans and scal ontologies along with concepts derived from the System Capabilities submodule of the Semantic Sensor Network (SSN) ontology. Although not a true ontology, the D-SI model is indispensable as it covers aspects essential to metrology and SI traceability.

In order to ensure that metrological traceability is a core part of our model, the description of observation values and metrological uncertainty will be carried out using the D-SI model [35] as before, while the OM ontology [36] will be used to model the physical quantities being measured. The traceability of the sensor measurements to the International System of Units (SI) as well as a systematic description of the physical units will thus be ensured. The SSN and SOSA ontologies will be used to model the sensing devices themselves. Moreover, the concepts defined in the SSN system capabilities module [43] will be used to ensure that the applicability of our ontology extends beyond physical sensors to soft-sensors and sensor aggregates. In order to provide a machine-interpretable description of the mathematical concepts required to compute a metric, the “Mathematical Object” class from the trans ontology defined in [31] will be used. The system capabilities extension of the SSN ontology, denoted by ssn-system, describes physical properties such as the survival and operating range as well as system properties such as drift, precision, resolution, and accuracy of a sensor. Through the use of ontologies, the concepts outlined in Section 2 can be systematically described while taking into account the relationships between them. The resulting merged ontology, which we call the qod ontology, is illustrated in Figure 3.

#### 3.1.1. Ontology Structure

An ontology based on the scheme outlined in Figure 1 needs to contain a formal description of the individual elements as classes as well as the relationships between them. In the following, we describe various representative classes and relationships of the merged ontology. Typically, a class or a relationship in an ontology is denoted by a prefix corresponding to the ontology name, followed by a colon and subsequently by the class/relationship name itself, i.e., prefix:name. We omit the prefix for entities that only belong to the new qod ontology and retain it for classes and relationships inherited from external ontologies. The main classes of the merged ontology derive from the concepts outlined in Section 3. These are:

- the ssn:System class imported from the SSN ontology representing the sensing system under investigation,
- the Indicator class that represents the abstract QoD indicator in question,
- the Metric class corresponding to the method used to calculate the QoD score with respect to a particular indicator and,
- the Interpretation class that describes the interpretation associated with a particular metric.

Similarly, the main relationships derive from those indicated in Figure 3. The Indicator class is related to the base system class by a hasIndicator object property. A System is not constrained to have a quality indicator. However, each Indicator must have at least one Metric, which in turn must have at least one Interpretation. The corresponding object properties in this case are hasMetric and hasInterpretation.

As in the case of the trans ontology, the mathematical building blocks needed to consistently represent the metrics introduced in Section 2.2 are objects from the trans:MathematicalObject class. In general, each metric can be defined by one or more mathematical objects. The relevant object property in this case is the trans:isExpressedBy. For instance, in the accuracy metric defined in Equation (1), if the distance D(.,.) is the Euclidean norm, the metric is given by a rational function (trans:RationalFraction) of the difference between the sensor value and the reference value $v_{n}-v_{n}^{\prime }$. Similarly, the consistency metric defined in Equation (3) is a piecewise continuous function and inherits from the corresponding OntoMath${}^{\mathrm{PRO}}$ object (mathematics:E1549). The topics covered by the merged ontology along with a corresponding motivation and illustrative sub-concepts are defined in Table 1. Each “has” relationship is necessarily accompanied by an “is-of” relationship. For instance, an indicator is related to a metric by the hasMetric relationship as well as an inverse isMetricOf relationship. The main axioms contained in the QoD ontology pertain to cardinality and type restrictions on the object and data properties. For instance, the Indicator class is asserted to be a subclass of the ssn:SystemProperty class in order to inherit its attributes. Furthermore, each Indicator is required to have at least one quality metric of type Metric which in turn is required to have at least one Interpretation. The interpretation is subsequently linked to at least one individual of the MathematicalObject class from the trans ontology in order to access the contained mathematical details. The aforementioned entities are connected by the relations described in Table 1. Each relation is accompanied by an inverse such that, for instance, a Metric is connected to an Interpretation by a hasInterpretation object property and inversely by an Interprets object property. The concepts added from external ontologies and data models are similarly listed in Table 2. The ontology corresponding to a given object is specified using an appropriate prefix (e.g., trans, ssn, sosa, om) The parameters corresponding to the individual mathematical objects are accompanied by the om:hasNumericalValue data property that assigns a content MathML string containing their numerical representations. Individual mathematical objects can also be connected to literal string datatypes via the trans:hasLiteralExpression data property. The sub-properties trans:hasMathMLExpression and trans:hasTeXExpression relate mathematical objects to MathML and T_E_Xstrings, respectively. qod ontology to literal datatypes. For example, the trans:hasMathMLExpression property relates MathML strings that describe the operation performed to compute the score of a metric to an individual of the Metric class.

## 4. Evaluation

The ontology merge was carried out using the Protegé [44] desktop tool and conflicts such as duplicated entries were manually resolved (The ontology files written in the RDF Schema are available on Github at https://github.com/PTB-M4D/QoD accessed on 27 September 2021). Methods for merging ontologies and evaluating the resulting merge [45] are a viable option, especially for very large ontologies. However, a manual check for duplicated entries was more feasible in the present case given the relatively small size of the qod ontology. A more thorough check for consistency was carried out using the HermiT (v. 1.38) reasoner on Protegé. In the following, we demonstrate the utility of the qod ontology with respect to providing a machine-interpretable representation of data quality by constructing a semantic description for a hypothetical use case. For this purpose we consider the x-axis angular velocity sensor of the MPU9250 inertial measurement unit [46] as an example. The MPU9250 is a system that consists of a three-axis accelerometer, a three-axis gyroscope, and a three-axis magnetometer. In order to illustrate the applicability of the qod ontology, the quality indicators of battery level and calibration data are considered. For the battery level, the percentage value is used as a metric, while the calibration data is quantified with two metrics, namely the measurement uncertainty and recency; cf. Section 2.2. The representation of the QoD assessment is achieved using the JSON-LD data format, with concepts from the proposed ontology mapped onto JSON objects. Finally, the scope of the ontology is elaborated further by formulating a series of competency questions.

### 4.1. JSON-LD Representation

The qod ontology described in Section 3.1.1 serves as a method to formally understand quality of data in sensor networks in terms of constituent base classes and the relationships between them. In order to apply the aforementioned formalism on real-world data, an appropriate method and format for encoding the entire QoD assessment is necessary. JSON-LD (JavaScript Object Notation for Linked Data) [47] is a promising format for this purpose. It is based on JSON [48], an open standard file and data-interchange format that uses human-readable text to store and transmit data objects. The JSON syntax is built on two main serializable structures:

- Unordered collections of comma-separated name/value pairs called *objects* enclosed in braces {}. Each name is necessarily a string which is followed by a colon and the value assigned to the name as “name”: value.
- Ordered lists of comma-separated values which are contained within square brackets []. Allowed values are strings specified in double quotes, numbers, booleans, null, or objects and arrays themselves. Objects and arrays can thus be nested.

White space characters like spaces, tabs, line breaks and carriage returns are ignored except within strings. Objects in JSON can be indefinitely nested and such a structure is conducive to representing a possibly elaborate QoD assessment containing both numerical scores and meaningful non-numerical metadata.

Linked data [49] in this context refers to structured data which is interlinked with other data so it becomes more useful through semantic queries. Structured data conforming to the qod ontology along with Internationalized Resource Identifiers (IRIs, [50]) provided for the base classes and relationships is an example of linked data. A JSON-LD representation is designed around a construct called the “context”, which is used to map IRIs to simpler terms. A context in the JSON-LD syntax that utilizes concepts from the qod ontology is given by:


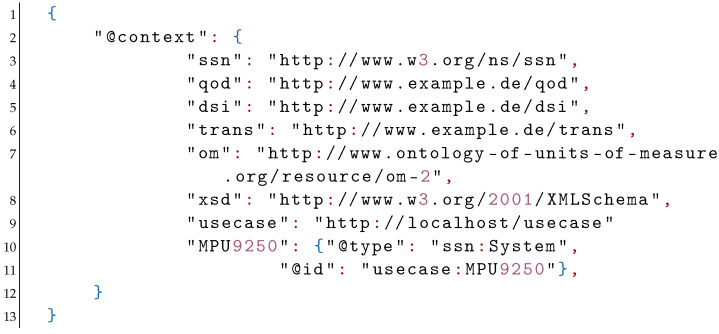


In the above example the IRI for the SSN (Semantic Sensor Network) ontology is mapped onto the string “ssn” and that of the qod ontology onto the string “qod”. The data corresponding to the use case is assumed to be locally stored and is referred to by the string “usecase”. Moreover, the om ontology (see Figure 2) and the XML schema are included as “om” and “xsd”. The sensor system under consideration is referred to as “MPU9250” and is specified to be of the type “ssn:System” and is given an id under the “usecase” namespace. The context can be included as-is in the JSON-LD file or it can be stored in an external file. We assume the latter case and subsequently import the context from an external file named “context.jsonld”. The description for the QoD indicator, metric, and interpretation for the battery level is given by:


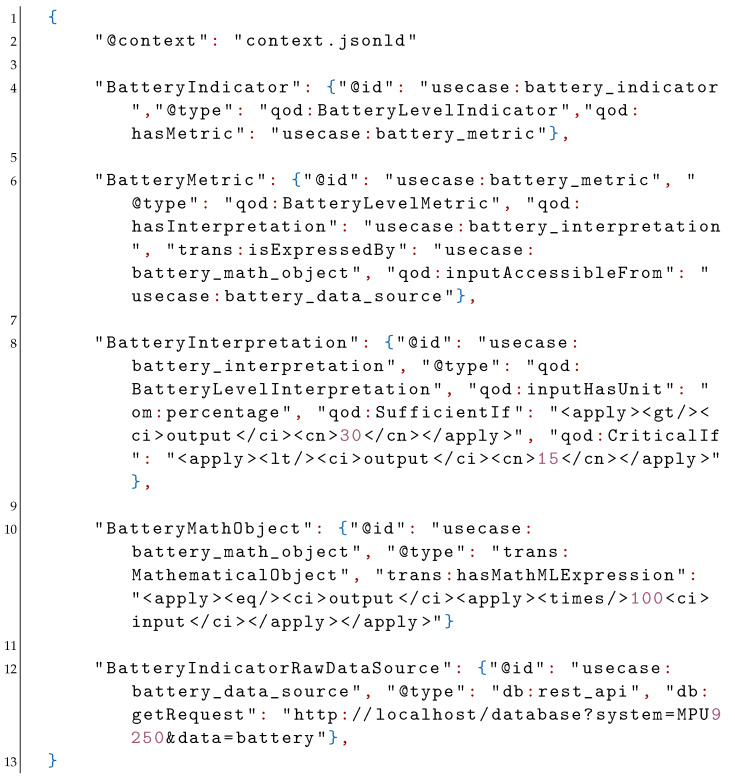


The first object after including the context file is assigned the id “usecase:battery_indicator” and is specified to be an object of the “qod:Indicator” class. It is then assigned a metric associated with the id “usecase:battery_metric”. The battery metric is specified to be of the type “qod:Metric” and is given an interpretation “battery_interpretation”. Moreover, the interpretation states that the input has a percentage defined using the qod:inputHasUnit relation and the om:percentage object from the OM ontology. The mathematical expression for the battery metric is given by “usecase:battery_math_object” and the raw data corresponding to the battery value is stated to be accessible at “usecase:battery_data_source”. The interpretation uses the data properties SufficientIf and CriticalIf to classify the battery level as sufficient if over 30 and critical if below 15 using MathML expressions. Similarly, the mathematical object is specified to be of type trans:MathematicalObject with a corresponding MathML expression that corresponds to the conversion of a given ratio input to a percentage value. Finally, the source of the battery data is available locally via a REST-API [51] and is named “usecase:battery_data_source”.

A similar description can be formulated if the calibration data is considered a quality indicator. In this case, the corresponding calibration indicator is specified to have two metrics: a measurement uncertainty metric and a recency metric (see Section 2.2).


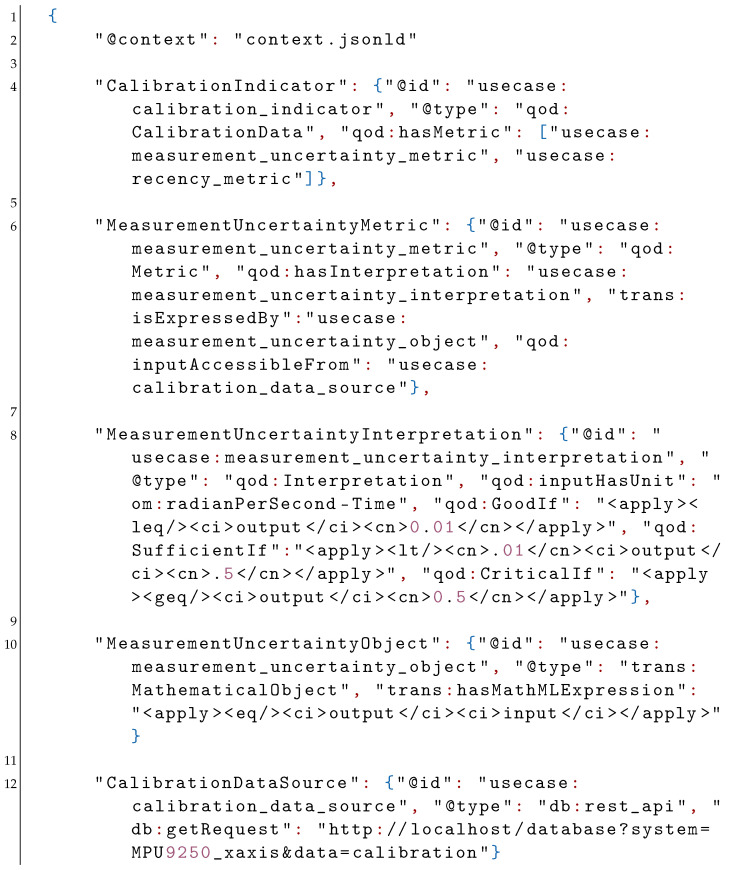


The measurement uncertainty metric is associated with a corresponding interpretation and is expressed by a measurement uncertainty object accessible from a specified data source. In contrast to the battery level, the measurement uncertainty interpretation categorizes the uncertainty as “good” if below 0.01, “sufficient” if between 0.01 and 0.5, and “critical” otherwise. Furthermore, the interpretation indicates that the input has angular velocity units (rad·s${}^{-1}$) via the OM ontology. The measurement uncertainty object is also of type MathematicalObject from the trans ontology and its MathML expression is a simple equality relation. In other words, the measurement uncertainty value received from the data source is directly used to interpret the QoD. As in the case of the battery level, the calibration data is available locally from the same source (e.g., a digital calibration certificate) via a REST-API and is named “usecase:calibration_data_source”.


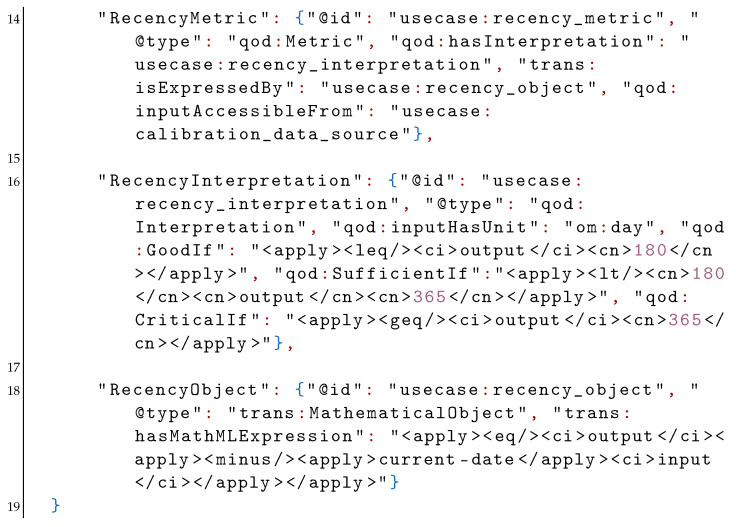


In a similar manner, the recency metric is associated with a recency interpretation and gets its data from the same calibration source as the uncertainty metric. The recency interpretation classifies the QoD as “good” if the calibration is newer than 180 days, “sufficient” if between 180 and 365 days, and “critical” if older. The interpretation also indicates that the input has the time unit om:day from the OM ontology. The recency object is now specified to be an XML duration object that is calculated by subtracting the calibration date from the current date. We have thus used the same data properties GoodIf, SufficientIf and CriticalIf to describe the QoD assessment for two different metrics for the same indicator.

### 4.2. Competency Questions

One of the key strengths of ontologies is that they enable automated processes to reason about rather coarse criteria for data processing and reach decisions without further human operator input. The qod ontology achieves this by providing a convenient way to add purpose-subjective interpretations of QoD metrics to an existing measurement system. A standard method to assess the ability of an ontology to provide answers to such vague questions is to use competency questions (CQs) [52]. CQs are typically represented as a set of questions and their respective answers are formulated in natural language such that the ontology is able to answer each question correctly. CQs also play a fundamental role in the development of an ontology by enabling the identification of the main elements and relationships in a domain [53]. In a system with multiple sensors of the type described in the previous section, the qod ontology is able to answer questions of the following type:

- Which sensors have a critical battery value?
- Which Metric was used to calculate the battery value?
- Which of two given sensors has a lower measurement uncertainty?
- Which sensors have not been calibrated recently?

**Figure 4:**
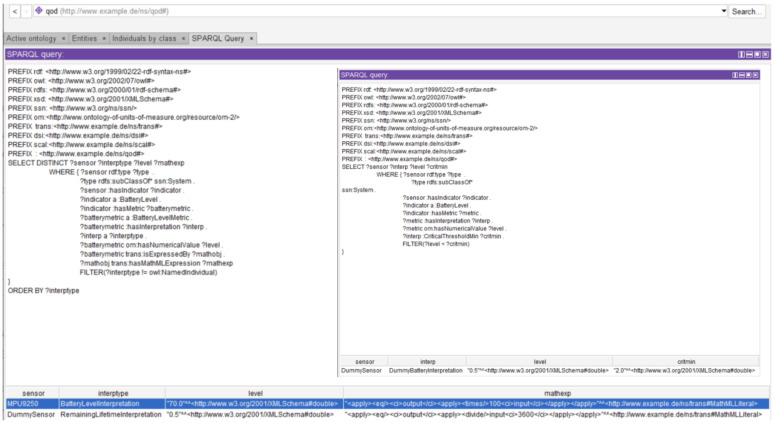
SPARQL queries corresponding to the battery level of sensors in the network. In the first query, the available sensors (MPU9250 and a dummy sensor) in the network with an indicated battery level are returned along with the corresponding interpretation-types. The battery level of both sensors and the mathematical expression used to convert the raw data values to interpretable numbers are also queried. Inset: The ontology is queried to find sensors with a critical battery level and the dummy sensor is returned due to its critical remaining lifetime.

In Figure 4, potential SPARQL queries corresponding to the first two of the above system-specific competency questions are shown. The first query returns the available sensors (MPU9250 and a dummy sensor) in the network with an indicated battery level. The type of interpretation corresponding to the battery level of the metrics is then queried and two different interpretations—battery level and remaining lifetime—are returned. Finally, the battery level of the sensors and the mathematical expression used to convert the raw data values to interpretable numbers (percentage and hours remaining in the present case) are queried and returned. In the inset, the ontology is queried to check which of the sensors have a critical battery level and the dummy sensor is returned as its remaining lifetime (0.5 h) is below the critical minimum threshold of 2 h.

The qod ontology can also answer general queries of the form

- Which types of Indicator are available for a given system
- Which types of Metric are available for a given Indicator?
- What is the MathematicalObject corresponding to the TimelinessMetric?

**Figure 5:**
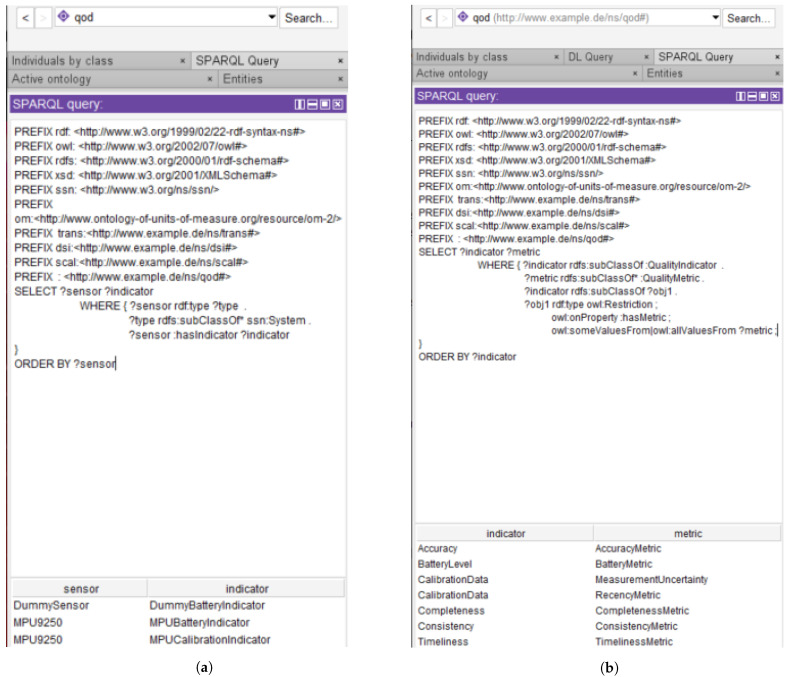
SPARQL queries and corresponding outputs applied on the qod ontology. (**a**) SPARQL query and result showing that the MPU9250 has both a battery level and calibration data as quality indicators, while the dummy sensor is only provided with a battery level indicator. (**b**) SPARQL query and result showing the different types of metrics available to the quality indicators in the ontology.

Example SPARQL queries along with their results corresponding to the first two questions are shown in Figure 5a,b. The first figure shows that the MPU9250 has two types of available quality indicators—a battery level and calibration data, while the dummy sensor is only provided with a battery level indicator. In the second case, we see the result of a system-independent query that shows which types of quality metrics are available for each indicator type. The calibration data, unlike the rest, can have both the recency of its calibration and its measurement uncertainty as metrics.

## 5. Conclusions

A basic scheme and an ontology for representing the quality of data in sensor networks have been presented. The concepts derived were then used to formulate a machine-interpretable description of QoD for a real-world use case. In Section 2 a semantic description of QoD was developed based on four main classes-systems, indicators, metrics and interpretations. A clear distinction was made between the metric which we defined as a method to calculate a score corresponding to a particular indicator, and the interpretation of the score itself. By integrating this distinction into our ontology, we emphasize the machine interpretability of our model at each level. The scheme introduced was consolidated into an ontology in Section 3 such that the proposed semantically expressive description of QoD was extended with relationships between the different concepts and between individual classes and datatypes. The qod ontology makes existing metrics explicit by relying on the trans ontology to describe the mathematical building blocks of the underlying computations. By including the SSN system capabilities module in our ontology, the inherent applicability of the model to soft sensors and sensor aggregates, in addition to physical sensors, was ensured. The constructed ontology was evaluated for the x-axis angular velocity sensor of the MPU9250 system in Section 4. A representation of the battery level and calibration data as quality indicators was presented along with corresponding metrics. For the battery level, a percentage value was used as a metric, while the recentness of the calibration and the measurement uncertainty were assigned as quality metrics for the calibration data. The QoD assessment for the aforementioned case was encoded in Section 4.1 using the JSON-LD format in order to take advantage of its hierarchical structure. The use of simple keys to refer to IRIs corresponding to ontology concepts greatly improves the human readability of the data format. Furthermore, a general evaluation of the qod ontology by means of a series of competency questions (CQs) was presented in Section 4.2. Future work will focus on the incorporation of QoD as additional inputs into machine learning algorithms that act on sensor network data. The key issues to be tackled in this regard is the influence of such higher level algorithms on QoD and finding appropriate methods for the assessment of QoD in soft and model-based sensors. The incorporation of events in the ontology in order to model the influence of changes in the sensor network structure, for instance with regard to sensor failure or the addition of new components, will also be a focus of future research.

## Figures and Tables

**Table 1:** Overview of new concepts in the qod ontology.

Concept	Motivation	Illustrative Sub-Concepts
Indicator	QoD indicator class	Accuracy, BatteryLevel, CalibrationDate
Metric	QoD metric that assigns a score to a given sensor for an indicator	Battery percentage, recency
Interpretation	Formal interpretation of the result of calculating a metric	Low/critical/sufficient battery level, calibration out of date
hasIndicator, isIndicatorOf	Relationship between a sensor and indicator	
hasMetric, isMetricOf	Relationship between an indicator and metric	
hasInterpretation, Interprets	Relationship between a metric and its interpretation	
inputHasUnit	Information regarding the units of the quantity used to calculate the metric	
inputAccessibleFrom	The data source from which information necessary to calculate the metric is accessible	

**Table 2:** Overview of external concepts the qod ontology.

Concept	Motivation	Illustrative Sub-Concepts
trans:MathematicalObject	Mathematical details of the metric	Array, Polynomial, Interval
trans:isExpressedBy	Relationship between an object (e.g., a metric) and a mathematical object	
trans:hasLiteralExpression	Attribute relating a mathematical object to a literal data type	trans:hasMathMLExpression, trans:hasTeXExpression
om:hasNumericalValue	Attribute assigning numerical values to mathematical objects, eg. coefficients of polynomials	
ssn:System	A unit of abstraction for pieces of infrastructure that implement Procedures.	sosa:Sensor, scal:CalibratedSensor
sosa:Sensor	Physical sensing device that observes a particular physical quantity	Accelerometers, barometers
sosa:Platform	A device or platform that hosts a sensor	Mobile phones
om:Measure	Assigning numerical values and physical units to model parameters[36]	Array, MeasureWithUncertainty
dsi:Uncertainty	Assigning uncertainties to model parameters [35]	StandardUncertainty, ExpandedUncertainty

## Data Availability

All data used in this publication is publicly available. Corresponding references are given in the paper.

## Abbreviations

The following abbreviations are used in this manuscript:

SSN	Semantic Sensor Network
SOSA	Sensor, Observation, Sample, Actuator
QoD	Quality of data
QoS	Quality of Sensing
IoT	Internet of Things
IIoT	Industrial Internet of Things
DCC	Digital Calibration Certificate
CQ	Competency Question
SI	Système international (d’unités)
VIM	Vocabulaire International de Métrologie
D-SI	Digital SI
IRI	Internationalized Resource Identifier
